# Platelet Proteasome Activity and Metabolism Is Upregulated during Bacterial Sepsis

**DOI:** 10.3390/ijms20235961

**Published:** 2019-11-27

**Authors:** Katharina Grundler Groterhorst, Hanna Mannell, Joachim Pircher, Bjoern F Kraemer

**Affiliations:** 1 Medizinische Klinik und Poliklinik I, Klinikum der Universität München, Marchioninistrasse 15, 81377 Munich, Germany; 2 Walter Brendel Centre of Experimental Medicine, University Hospital, Ludwig-Maximilians-University, Marchioninistr. 27, 81377 Munich, Germany; Hanna.Mannell@med.uni-muenchen.de; 3 Biomedical Center, Ludwig-Maximilians-University, Großhaderner Str. 9, 82152 Planegg, Germany; 4 DZHK (German Center for Cardiovascular Research), Partner Site Munich Heart Alliance, 80802 Munich, Germany

**Keywords:** proteasome activity, platelets, sepsis, bacteria

## Abstract

Dysregulation of platelet function can contribute to the disease progression in sepsis. The proteasome represents a critical and vital element of cellular protein metabolism in platelets and its proteolytic activity has been associated with platelet function. However, the role of the platelet proteasome as well as its response to infection under conditions of sepsis have not been studied so far. We measured platelet proteasome activity by fluorescent substrates, degradation of poly-ubiquitinated proteins and cleavage of the proteasome substrate Talin-1 in the presence of living *E. coli* strains and in platelets isolated from sepsis patients. Upregulation of the proteasome activator PA28 (PSME1) was assessed by quantitative real-time PCR in platelets from sepsis patients. We show that co-incubation of platelets with living *E. coli* (UTI89) results in increased degradation of poly-ubiquitinated proteins and cleavage of Talin-1 by the proteasome. Proteasome activity and cleavage of Talin-1 was significantly increased in α-hemolysin (HlyA)-positive *E. coli* strains. Supporting these findings, proteasome activity was also increased in platelets of patients with sepsis. Finally, the proteasome activator PA28 (PSME1) was upregulated in this group of patients. In this study we demonstrate for the first time that the proteasome in platelets is activated in the septic milieu.

## 1. Introduction

Sepsis is a life-threatening disease caused by a dysregulated inflammatory host response, often induced upon a systemic bacterial infection. In this context, *E. coli* is a frequent cause of sepsis. Apart from an exaggerated systemic inflammatory response, a pro-coagulant and pro-thrombotic state is present during sepsis and the uncontrolled activation of platelets can contribute to the progression of the disease [1]. Recent work has demonstrated that platelets also possess a functional proteasome [2] and others and we have shown that platelet function is associated with proteolytic regulation of proteins by the proteasome [3,4]. Moreover, the expression as well as the proteolytic activity of the proteasome were shown to be increased in muscle tissue during sepsis [5,6,7]. However, proteasome activity has not yet been studied in platelets during sepsis. 

The proteasome represents a critical element for protein processing in human cells and is crucial for protein degradation, turnover and antigen presentation [8]. Proteins designated for proteasome processing are tagged with ubiquitin to be unfolded and identified by the proteasomal catalytic subunits [9]. Especially, in platelets, as anucleate cells, the proteolytic cleavage of proteins is an important mechanism for regulation of their cellular functions [10]. Indeed, proteasomal activity was shown to be important for platelet aggregation and thrombosis formation in vitro and in vivo and interestingly, these physiological processes could be efficiently prevented by proteasome inhibition [2,3,4,10,11]. Moreover, by studying the proteasomal cleavage of proteins involved in cytoskeletal regulation, such as Filamin A and Talin-1, our group was able to identify a link between the proteasome and NFκB in the regulation of collagen-induced platelet aggregation [3]. During inflammatory conditions, additional proteasomal subunits (PSME1 and PSME2) are expressed and form an immunoproteasome together with subunits of the conventional proteasome [12]. Apart from its important role in antigen presentation by MHC class I molecules, the immunoproteasome has been shown to exhibit a higher proteolytic activity and to prevent cellular damage during inflammation [13]. Of note, a functional immunoproteasome as well as the capacity to process and present antigens is present also in platelets [14,15]. Malfunction of the proteasome has been associated with several disease processes [16]. However, our knowledge about its role and function in platelets, especially under disease conditions, is still scarce.

In this study, we therefore investigated the activity of the proteasome in platelets in the septic milieu using living *E. coli* in vitro and in sepsis patients. We observed an upregulation of the immuno-proteasome subunit and activator PA28 (PSME1) in platelets from sepsis patients and increased processing of polyubiquitinated proteins as well as the proteasome substrate Talin-1 under conditions of sepsis. Proteasome activation was more pronounced when platelets were exposed to pathogenic *E. coli* (UTI89) expressing the exotoxin α-hemolysin compared to toxin-negative *E. coli* strains. Our novel data demonstrate that the proteasome in platelets responds to the septic environment and is upregulated in patients with sepsis.

## 2. Results

### 2.1. Platelet Proteasome Activity and Protein Metabolism is Increased in the Septic Milieu

**Figure 1:**
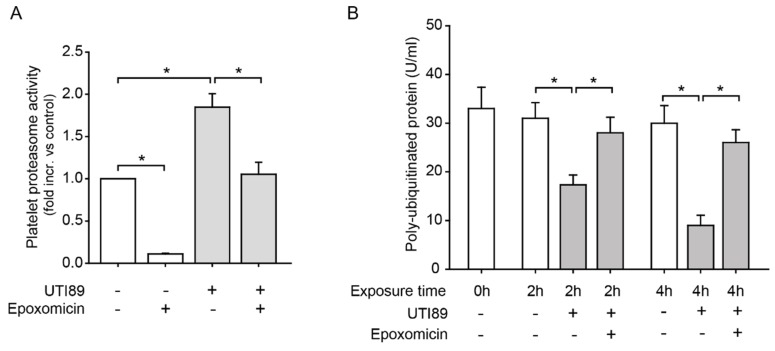
Bacteria induce proteasome activation and increased protein degradation in human platelets. (**A**) Co-incubation of platelets with living *E. coli* (UTI89) for 4 h induced a significant increase in platelet proteasome activity measured by fluorescent substrate cleavage, compared to control platelets (*p < 0.05, *n* = 3). Increased activation of the proteasome was effectively reversed with the proteasome inhibitor epoxomicin (10 µM; *p < 0.05, *n* = 3). (**B**) Platelet coincubation with living *E. coli* (UTI89) led to accelerated degradation of polyubiquitinated proteins (U/mL) in platelets after 2 and 4 h of incubation, as assessed by ELISA. This was effectively inhibited by proteasome inhibition (epoxomicin, 10 µM; *p < 0.05, *n* = 3).

As systemic *E. coli* infection is a frequent cause of sepsis, we were first interested in whether *E. coli* affects platelet proteasome activity. Incubation of isolated human platelets with the pathogenic *E. coli* strain UTI89 led to increased proteasome activity in vitro. This effect was specific, as it was effectively inhibited by the proteasome inhibitor epoxomicin (Figure 1A). Poly-ubiquitinated proteins, which represent proteins marked for proteasomal processing, were excessively degraded over time during coincubation with *E. coli* UTI89. This process was equally inhibited by treatment with epoxomicin (Figure 1B). 

### 2.2. E. coli Exotoxin α-Hemolysin (hlyA) may be a Contributing Factor to Increased Proteasome Activity in Platelets

**Figure 2:**
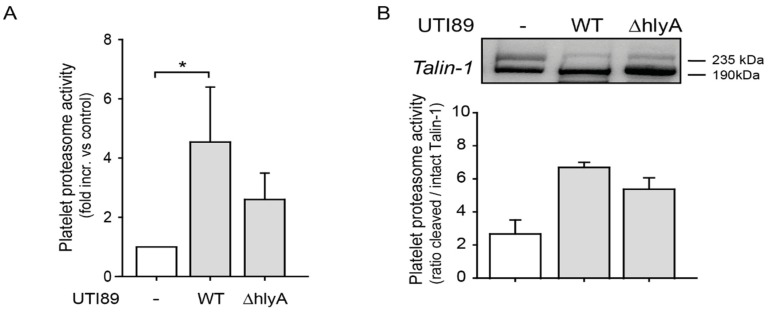
α-hemolysin expression in *E. coli* enhances platelet proteasome activation. (**A**) Platelet proteasome activity was enhanced upon incubation (4 h) with α-hemolysin expressing *E. coli* (UTI89 WT), whereas incubation with *E. coli* lacking functional α-hemolysin (UTI89 ΔhlyA) showed a trend towards less proteasome activation, as assessed by fluorescent peptide cleavage (*p < 0.05, *n* = 5). (**B**) The proteasome substrate Talin-1 was increasingly cleaved during coincubation with *E. coli* (UTI89) (ratio of 190 kDa to 235 kDa fragments) compared to control platelets. Increase of cleavage was less with the α-hemolysin negative strain UTI89 ΔhlyA compared to platelet control (*n* = 2).

*E. coli* α-hemolysin is a potent exotoxin, which can activate proteases in a calcium-dependent fashion [17,18]. Platelet proteasome activity, assessed by fluorescent substrate cleavage, was significantly enhanced in the presence of *E. coli* UTI89 (Figure 2A). Effects were less pronounced during coincubation with *E. coli* strains, where the α-hemolysin gene was knocked-out (UTI89 ΔhlyA). Typical cleavage of the proteasome substrate Talin-1 by the proteasome from a 235 kDa fragment to a 190 kDa fragment was also increased in the presence of *E. coli* UTI89 (ratio 190/235 kDa). This effect was again weaker when platelets were coincubated with the hlyA-negative *E. coli* strain UTI89 ΔhlyA (Figure 2B). 

### 2.3. Platelet Proteasome Activity is Increased in Platelets from Sepsis Patients 

**Figure 3:**
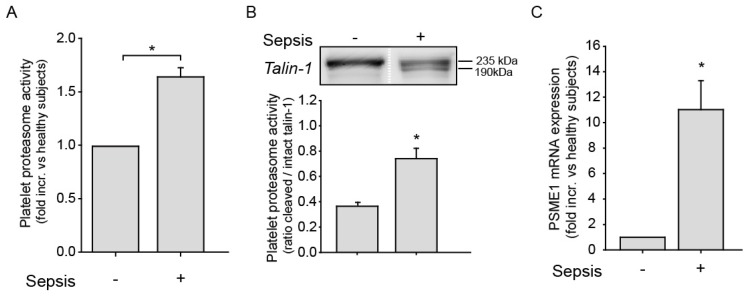
Platelets proteasome activity is increased in platelets of sepsis patients. (**A**) Platelet proteasome activity is increased in patients with sepsis (*p < 0.05, *n* = 7) compared to healthy controls (*n* = 9). (**B**) Cleavage of the proteasome substrate Talin-1 from a 235 to a 190 kDa fragment is significantly increased in platelets of the sepsis population (*n* = 5) compared to controls (*n* = 3; *p < 0.05). Proteasome activity was expressed as a ratio of cleaved 190 kDa to intact 235 kDa Talin-1 products by immunoblotting. The protein bands from a healthy control and a sepsis patient in the western blot image are derived from the same blot but cropped out and placed next to each other (white dotted line) for illustrative purposes. (**C**) mRNA expression analysis by real-time PCR revealed that the proteasome activator PA28 (PSME1) was overexpressed in platelets of sepsis patients compared to healthy individuals (*n* = 4, *p < 0.05).

Having observed that pathogenic *E. coli* enhances platelet proteasome activity, we next studied the platelet proteasome in patients with bacterial sepsis. Clinical information on sepsis patients is shown in Table 1. As seen in Figure 3A, proteasome activity in platelets from sepsis patients was significantly upregulated compared to healthy controls. In addition, cleavage of the proteasome substrate Talin-1, expressed as a 190 to 235 kDa ratio, was also significantly increased in platelets of sepsis patients compared to controls (Figure 3B). As the immunoproteasome has been shown to be upregulated upon inflammatory conditions, we next investigated immunoproteasome activator PA28 (PSME1) expression in platelets from sepsis patients. mRNA expression analysis using real-time PCR revealed an upregulation of PA28 (PSME1) in platelets of sepsis patients compared to controls (Figure 3C).

### 2.4. Clinical Characteristics of Sepsis Patients

We have summarized clinical characteristics of sepsis patients in Table 1.

## 3. Discussion

In the present study, we demonstrated for the first time that the proteasome of platelets was not static but responded to environmental stress conditions, such as a septic milieu, by increasing proteasome activity and protein metabolism. We know that the proteasome is activated in muscle tissue and in muscle wasting in sepsis [6,7], but in platelets its activity and response under septic conditions has not been studied so far. In vitro, we demonstrated that exposure of platelets with living *E. coli*, which is frequently identified in blood cultures of sepsis patients, increased platelet proteasome activity and subsequently resulted in increased degradation of poly-ubiquitinated proteins in platelets. This metabolic activation was effectively inhibited with the proteasome inhibitor epoxomicin. Polyubiquitination is a characteristic mechanism for labeling proteins for proteasomal processing and thus levels of polyubiquitinated proteins represent a good marker for proteasomal activity. More precisely, we showed that the proteasome substrate Talin-1 is excessively cleaved by the proteasome from a 235 to a 190 kDa fragment after co-incubation with *E. coli* (UTI89). Talin-1 has been shown to be a classical target of the proteasome and functional aspects of platelet aggregation and shape change have been associated with proteolytic regulation by the proteasome [3,4]. Talin-1-derived peptides were found to be presented on HLA-1 molecules on the platelet surface, which also confirms that Talin-1 is processed by the (immuno)-proteasome of platelets (unpublished observations). To investigate underlying mechanisms how septic processes may affect the proteasome, we compared pathogenic *E. coli* strains expressing functional α-hemolysin (UTI89) to strains lacking α-hemolysin (UTI89 ΔhlyA) [19,20]. α-hemolysin is a strong pathogenic factor of bacteria that can induce cell death in platelets as previously shown [17]. By measuring platelet proteasome activity by fluorescent substrate and Talin-1 cleavage during coincubation with living *E. coli*, we observed that the increase of proteasome activity seemed to be more pronounced in wild-type α-hemolysin positive UTI89 strains compared to strains not expressing α-hemolysin (UTI89 ΔhlyA). Although this needs further confirmation, the results suggest that α-hemolysin may be one of the pathogenic factors mediating the activation of the platelet proteasome. As previously shown for calpain [17], this process likely involves calcium-dependent activation of proteasomal subunits to increase proteolytic activity, although the precise mechanism remains to be elucidated in future studies. Finally, to study platelet proteasome activity in a complex sepsis environment, we compared platelets from patients with bacterial sepsis to healthy patients on an mRNA expression level ex vivo. Using real-time PCR we observed upregulation of the proteasome activator PA28 (PSME1) in platelets specifically in the sepsis population in line with increased proteasome activity. This finding was of particular interest, as PA28 (PSME1) is primarily an activator of the immunoproteasome that processes peptides for antigen presentation and which is activated during inflammation [5]. The presented data support our concept that the proteasome in platelets is involved in cellular adaptation to inflammatory environmental stress, such as sepsis, on different levels. PA28 (PSME1) encodes for the 11S proteasomal subunit that replaces the 19S regulatory subunit when the immunoproteasome is assembled. PA28 (PMSE1) is expressed in platelets at the mRNA level [15] and protein level [21] as previously shown. Interestingly, the complete machinery to process and present antigens on the cell surface via MHC-I is present in platelets, which underscores that platelets play an active role in the immune defense [14,15]. As an example, previous work shows that HLA-1 expression in platelets is altered during infection with dengue virus [22]. Supporting our in vitro findings, we observed increased platelet proteasome activity in platelets of patients with sepsis by fluorescent substrate cleavage as well as significantly increased cleavage of the proteasome substrate Talin-1 in the sepsis population ex vivo. Although this finding supports the observation that *E. coli* induces proteasomal proteolytic activity, we cannot exclude that the inflammatory environment per se additionally contributes to increased proteasome activity in sepsis patients. It also still remains to be investigated, which specific effects an increased proteasome activity has on platelet function in sepsis. Nevertheless, our collected data implied that platelet proteasome activity was affected both in terms of activity and function but also on an mRNA expression level during sepsis.

## 4. Materials and Methods 

### 4.1. Materials

Talin-1 antibody was purchased from Cell Signaling, Danvers, USA, and horseradish peroxidase-conjugated secondary antibody was from Merck Millipore (Billerica, MA, USA). Epoxomicin was purchased from EMD Biosciences (LaJolla, CA, USA). All other chemicals were purchased from Sigma Aldrich (Darmstadt, Germany).

### 4.2. Bacteria

*E. coli* bacteria of the α-hemolytic strain (hlyA) UTI89 [19] as well as the *E. coli* strain with deletion of hlyA (UTI89 ΔhlyA) [20] were a kind gift from Dr. Matthew Mulvey, Department of Pathology at the University of Utah (Salt Lake City, UT, USA). Similar to recent work [17], bacteria were expanded on blood agar plates overnight at 37 °C until they reached a stationary growth phase. Bacteria were then resuspended in phosphate-buffered saline (PBS) and their concentration was determined by colorimetry (VITEK Colorimeter, bioMerieux, Inc., Durham, NC, USA). Prior to experiments, *E. coli* bacteria were resuspended in M199 culture. For every experiment, the soluble agonists or bacteria were incubated in the presence of freshly isolated platelets (1 × 10^8^ platelets at bacteria to platelet ratio of 1:30) for 4 h in M199 culture media under cell culture conditions.

### 4.3. Platelet Isolation and Preparation 

As previously described [3,17,23], washed platelets were freshly isolated from healthy human subjects and patients with bacterial sepsis, who consented to participate in the study. All studies involving patients and human cells were performed in accordance with the declaration of Helsinki and were approved by the local ethics committee (No. 290-11, approved at 12 July 2011). For inhibitor studies of the proteasome, platelets were incubated with epoxomicin (10 µM) for the indicated time in warm M199 media under cell culture conditions (5% CO_2_, 37 °C).

### 4.4. RNA Isolation

Platelets (1 × 10^9^) were isolated from healthy donors and patients with bacterial sepsis and were exposed to CD45 positive selection, which effectively depletes contaminating leukocytes as previously described [24]. Cells were lysed in Trizol (Invitrogen, Carlsbad, CA, USA) and glycogen was added to the aqueous phase before precipitation with isopropanol to optimize RNA yields. The RNA was treated with DNAse (DNA free Kit, Ambion, Austin, TX, USA), precipitated with ethanol and dissolved in 12 µL of RNAse-free water. RNA (1 µg) was used to generate cDNA to characterize the expression of PSME1. Integrin αIIb was used as a positive control for platelet-specific RNA. The relative abundance of the PSME1 RNA was measured by real-time PCR. Primer sets for these studies were as follows: PSME1, forward-GAAGCCAACTTGAGCAATCTGA, reverse-AGCCTTCTAGCTTGGTGTGGAG; and β2-Microglobulin, forward- ACACTATTCTAGCAGGAGGGTTGG, reverse- CAGGGCTCAGTCTCTTTATTAGGC. 

### 4.5. Detection of Platelet Proteasome Activity

Proteasome activity was measured using a proteasome activity assay with fluorescent substrate according to the manufacturer’s instructions (Millipore, Billerica, MA, USA) and as previously described [3]. For experiments where platelets were exposed to living *E. coli* bacteria (see above), cells were pelleted and lysed after 4 h of incubation. As a negative control equal amounts of bacteria were incubated under identical conditions, pelleted, lysed and incubated with the proteasome substrate. Platelet proteasome activity (relative fluorescent units, RFU) was detected at 4 h under cell culture conditions at 450 nm using a fluorescence microplate reader (Tecan Group, Mannedorf, Switzerland).

### 4.6. Quantification of Polyubiquitinated Protein in Human Platelets

After incubation with living *E. coli* or the proteasome inhibitor epoxomicin, cells were pelleted and lysed in 200 µL of ubiquitinated protein lysis buffer according to the manufacturer’s protocol (human poly-ubiquitinated protein ELISA, MBL International, Woburn, MA, USA). Platelet lysates were diluted 1:200 in the provided dilution buffer and then added to the ELISA plate. ELISA was carried out according to the manufacturer’s instructions. Quantitative changes of total poly-ubiquitinated protein (U/mL) in platelets were measured at 450 nm with an ELISA plate reader (BMG LabTec, Worchester, MA, USA). 

### 4.7. Immunoblotting

Immunoblotting for Talin-1 was performed as previously described [3]. In brief, pellets were lysed in cell lysis buffer (Cell Signaling, Danvers, MA, USA) containing 1 mM PMSF. After centrifugation at 10,000 g for 5 min at 4 °C, protein quantification was performed through the bicinchoninic acid assay (ThermoScientific, Waltham, MA, USA) according to the manufacturer’s protocol. Equal volumes of sample were separated by SDS-PAGE using 10% gels and blotted onto PVDF membrane. Membranes were blocked in 5% milk and dissolved in Tris buffered saline with 0.1% Tween and were subsequently incubated with the primary antibody against Talin-1 (1:1000 dilution) at 4 °C overnight. Membranes were washed and incubated with horseradish peroxidase-conjugated secondary antibodies at room temperature for 1 h. Enzymatic activity was detected with a chemiluminescence detection kit according to the supplier’s protocol and recorded with a digital camera (Hamamatsu Photonics, Hamamatsu City, Japan). Protein band density measurements for Talin-1 235 kDa and 190 kDa fragments were performed with Image J software. For evaluation of proteasomal cleavage of Talin-1, the ratio 235 kDa/190 kDa was calculated.

### 4.8. Statistical Analysis

All data are presented as means ± SEM. Statistical analyses were performed with Sigma Plot 10.0. For comparisons between two groups of normal distributed data, the student’s *t*-test was used. For the comparison of two groups without normal distributed data, a rank-sum test was performed. For multiple comparisons between groups of normal distributed data, the one-way analysis of variance (1-way ANOVA) was used. Differences were considered significant at an error probability level of *p* < 0.05.

## 5. Conclusions

In summary, our study provided novel evidence that the proteasome of platelets responded to the septic environment by increased proteasome activity, increased proteolytic cleavage of proteasome substrates such as Talin-1 and upregulation of (immuno)proteasome activator PA28 (PSME1). Although we still know little about the precise function of the proteasome in platelets, it appeared that the proteasome was an important element in the response to severe cellular stress situations such as sepsis. Future studies will have to show if this response is simply a matter of increased metabolic protein turnover required during systemic inflammation or if it serves the purpose of anti-pathogenic defense strategies, antigen processing, hemostasis or all of them. This study, however, provides a basis to further characterize the precise role and regulation of the proteasome in platelets in complex disease processes, such as sepsis.

## Figures and Tables

**Table 1:** Clinical characteristics of seven patients with bacterial sepsis are shown. Patients were diagnosed with bacterial sepsis based on clinical presentation and positive bacterial culture results. Markers of inflammation including leukocyte count, C-reactive protein (CRP) and procalcitonin were markedly elevated. SOFA score (sequential organ failure assessment) showed multi-organ dysfunction and all patients were treated with antibiotics. Data is presented as median and (interquartile range (IQR)), n = 7.

Clinical Characteristics of Seven Patients with Bacterial Sepsis	
Age	63 [IQR 49; 69]
Leukocyte count (1000/µL)	16 [IQR 11.0; 21.0]
CRP level (mg/dl)	25 [IQR 19.5; 28.5]
Procalcitonin (ng/mL)	5.5 [IQR 4.0; 9.8]
Positive bacterial culture	7 of 7
Antibiotic treatment	7 of 7
SOFA score	9 [IQR 6; 11]
